# Diagnostic performance analysis of the Integrated Care for Older People (ICOPE) screening tool for identifying decline in intrinsic capacity

**DOI:** 10.1186/s12877-023-04180-x

**Published:** 2023-08-23

**Authors:** Fei Lu, Jiaojiao Li, Xiaohong Liu, Shuo Liu, Xiaohong Sun, Xueying Wang

**Affiliations:** 1 Department of Geriatrics, Peking Union Medical College, Chinese Academy of Medical Sciences, Peking Union Medical College Hospital, No. 1 Shuai Fu Yuan, Dong Cheng District, Beijing, China; 2 Yanyuan Rehabilitation Hospital, No.2, Jingrong Street, Nanshao Town, Changping District, Beijing, China

**Keywords:** Diagnostic performance, Intrinsic capacity, ICOPE screening tool, Older people

## Abstract

**Background:**

Intrinsic capacity (IC) is a comprehensive indicator of an individual’s positive attributes. The World Health Organization (WHO) recommends a two-step approach to assess IC decline among older people. The first step involves the used of the integrated care for older people (ICOPE) screening tool to identify potential issues, and the second step involves using detailed assessments for confirmation. This study aimed to assess the diagnostic performance of the ICOPE screening tool as a simple preliminary screening to identify IC decline among community-dwelling older people, which has been rarely reported in China.

**Methods:**

This cross-sectional study included 228 community-dwelling older individuals aged ≥ 75 (mean age, 84.0 ± 4.4 years; 131 [57.5%] females) who completed the IC evaluation according to the WHO IC assessment pathway. The diagnostic performance of the ICOPE screening tool was calculated using a 2 × 2 table and a receiver operating characteristic curve.

**Results:**

The proportion of possible IC decline identified by the ICOPE screening tool was 79.4%, whereas the actual IC decline assessed by the detailed assessment was 73.2%, mainly in locomotion. The ICOPE screening tool showed sensitivity and specificity of 94.6% and 62.3%, respectively, with an overall diagnostic accuracy of 86.0%. The diagnostic effectiveness of the ICOPE screening tool was 0.91 (95% confidence interval: 0.87–0.95, *p* = 0.020). Except for the sensory dimension, the sensitivity of the ICOPE screening tool for diagnosing impairments in each dimension of the IC was the highest in the cognition domain (100%) and the lowest in the vitality domain (51.3%), whereas the specificity was the highest in vitality (94.7%) and the lowest in cognition (55.6%).

**Conclusions:**

The ICOPE screening tool exhibits high sensitivity and can be used as an IC screening tool in community-dwelling older people. However, further improvements are needed in the vitality dimension of the ICOPE screening tool to enhance its sensitivity in identifying individuals at risk of malnutrition.

## Background

The aging global population is constantly increasing. As a populous country, China faces a severe aging challenge. According to the National Bureau of Statistics of China’s 2022 investigation on population changes [1], the proportion of people aged 60 years and above has reached 19.8%, with those aged 65 years and above accounting for 14.9%, indicating that China has become an aging society. Those born during the second baby boom, between 1962 and 1975, will gradually enter retirement by 2023, further increasing the burden of aging in China. In 2022, the Lancet published a report on China’s path to healthy aging [2], which showed that its life expectancy has gradually approached that of other developed countries.

In 2021, the World Health Organization (WHO) proposed the “Decade of Healthy Aging 2020–2030” [3], which involves the following four areas: (1) providing person-centered integrated care to meet the needs of older people; (2) changing attitudes and behaviors towards aging; (3) supporting community interventions to maintain the intrinsic capacity (IC) of older people [4] and promote functional ability; and (4) providing long-term care for older people in need. Therefore, the concept of healthy aging implies that the medical model for older people should shift from a disease-centered to a function-centered approach [5]. IC is the basis of functional turning.

The concept of IC is an evolution of the International Classification of Functioning, Disability, and Health framework proposed by the WHO in 2001 [6]. Within this framework, the health condition (disease) construct is inadequate to define IC. Only the functional domain aligns with IC, which was proposed in 2015 by the WHO [4]. The WHO [4] defined the concept of IC as a combination of all the mental and physical capacities that an individual can access at any time.

In 2018, Cesari et al. [7] reviewed the literature and proposed the following five dimensions of IC: locomotion, cognition, vitality, psychology, and sensory (hearing and vision). Visual and hearing impairments can affect older people’s activities and cognition, and the presence of both can exacerbate these effects [7]. Therefore, they are generally considered as two capacities within the sensory dimension. Beard et al. [8] verified the validity of this multidimensional model based on the China Health and Retirement Longitudinal Study and found that the individual’s IC could reflect functional ability. Yu et al. [9] in Hong Kong also supported the construction of the five domains of IC using structural equation models. In 2019, the WHO proposed the person-centered assessment and pathways in primary care [10], which contains 5 steps. The first step involves the use of the integrated care for older people (ICOPE) screening tool to identify potential IC decline issues, and the second step involves the use of detailed assessments to confirm individuals with actual IC decline. Based on this assessment, personalized interventions [10] can be provided to older people with IC decline in step 3. Steps 4 and 5 involve monitoring care plans and supporting caregivers, respectively.

However, what is the diagnostic performance of the ICOPE screening tool in detecting IC decline? There are two published studies, one from Hong Kong, China [11], and the other from Spain [12]; both of them indicate that the ICOPE screening tool has high sensitivity but varying screening effectiveness across different dimensions. Additionally, no relevant studies have been conducted in mainland China.

Therefore, this study aimed to evaluate diagnostic performance, which includes the sensitivity, specificity, and diagnostic accuracy of the ICOPE screening tool to determine whether the screening tool is suitable for community-dwelling older people using step 2 of the detailed assessment of IC as the gold standard.

## Methods

### Study design and participants

This study used the baseline data of a previous cohort study [13]. This cohort study primarily aimed to explore the trajectory of IC in older people and its predictive value for adverse outcomes. The external environment also influences the functional performance of older people and the occurrence of adverse outcomes [14]. Therefore, to reduce the confounding effects of the external environment and support systems and better explain the impact of individual differences in IC, this study selected residents of a relatively consistent external environment in an elderly friendly community (Taikang Yanyuan) in Beijing as the research participants. This study cohort included community-dwelling older people continuously from July–September 2018. The inclusion criteria were as follows: individuals aged ≥ 75 years and those who had completed the IC assessment. The exclusion criteria were as follows: individuals with acute illness within three months prior to enrollment, including but not limited to acute coronary syndrome, pulmonary infection, and stroke.

### Measurements

Sociodemographic data, such as age, sex, height, weight, and education level, were collected. Polypharmacy refers to the use of ≥ 5 medications, including prescription and non-prescription drugs [15].

The Cumulative Illness Rating Scale for Geriatrics (CIRS-G) [16] was used to assess comorbidities. This scale includes the distribution of diseases in 14 systems. Each system is classified into 5 levels based on disease severity, with scores ranging from 0–4. When multiple diseases exist within the same system, the score is based on the highest severity level. The scores of each item are summed to obtain a total score.

Basic activities of daily living (ADL) were evaluated using the physical self-maintenance scale (PSMS) [17], which evaluates basic self-care activities, such as toileting, grooming, feeding, dressing, physical ambulation, and bathing. Each item was scored as 1 point, with a total score ranging from 0–6. A score of < 6 indicated a decline in ADL. Furthermore, instrumental activities of daily living (IADL) were assessed using the Lawton IADL scale [17]. This scale evaluates activities such as telephone use, food preparation, responsibility for own medications, financial management, shopping, housekeeping, laundry, and mode of transportation. Each item was scored as 1 point, with a total score ranging from 0–8. A score of < 8 indicated a decline in IADL.

### Intrinsic capacity

This study was based on the WHO IC assessment pathway [10] (Table 1), with step 1 being an IC screening evaluation of the participants using the ICOPE screening tool. The total score ranged from 0–9 points, and a score of ≥ 1 indicated a possible IC decline. Step 2 involved a detailed IC evaluation [18–21] of IC to identify older people with actual IC decline. The total score ranged from 0–6 points, and a score of ≥ 1 indicated actual IC decline. In this study, visual and hearing impairments were defined as impairments impacting daily life during screening and detailed assessment.

**Table 1:** 2-Step intrinsic capacity assessment: ICOPE screening tool and detailed assessment^1^

IC dimensions	ICOPE screening tool	Detailed assessments
Locomotion	Five times sit-to-stand test ≥ 14 s	SPPB ≤ 9 score
Vitality	Loss more than 3 kg of weight in the past 3 months	MNA-SF ≤ 11 score
	Loss of appetite in the past 3 months	
Cognition	Cannot recall three words	MMSE < 24 score
	Disorientation in time or space	
Psychology	Depressed or hopeless in the past two weeks	GDS-15 ≥ 5 score
	Lost interest in activities in the past two weeks	
Sensory (vision)	Vision deteriorated and affected daily life	Same
Sensory (hearing)	Hearing deteriorated and affected daily life	Same

*Abbreviations*: ^1^If any problem in the table satisfied is scored 1. The total score of the ICOPE screening tool ranges from 0–9 points, and a score of ≥ 1 indicates possible IC decline. The total score of detailed IC assessments ranges from 0–6 points, and a score of ≥ 1 indicates actual IC decline. *IC* Intrinsic capacity, *SPPB* Short physical performance battery, *MNA-SF* Short-form mini-nutritional assessment, *MMSE* Mini-mental state examination, *GDS-15* 15-item geriatric depression scale

### Statistical analysis

Baseline data were collected, and normally distributed data were expressed as mean ± standard deviation (x ± s), while non-normally distributed data were expressed as median (interquartile range) [M (IQR)]. Quantitative data were presented as constituent ratios, rates, and absolute numbers. The validity of the ICOPE screening tool for detecting IC decline was calculated using a four-fold table and the receiver operating characteristic curve. All data were analyzed using SPSS26.0 for Windows (IBM Corp. Armonk, NY, USA).

## Results

### Baseline characteristics

Overall, 228 older people were included in this study (Table 2), with a mean age of 84.0 ± 4.4 years. Of them, 131 (57.5%) were female, and 225 (98.7%) had an education level of high school or above. The CIRS-G score was 5 (3–7) points. The ADL score was 6 (6–6) points, indicating a good functional status. Furthermore, the ICOPE screening tool score was 2 (1–4) points, and 181 (79.4%) participants were identified as having a possible IC decline. In the detailed evaluation of step 2 IC, the score was 1 (0–2), indicating that 167 (73.2%) older people had an actual IC decline.

**Table 2:** Baseline characteristics of 228 participants

Variable	Total
	*n* = 228
Age, mean (SD), years	84.0 ± 4.4
Female, n (%)	131 (57.5)
BMI, mean ± SD, kg/m^2^	24.2 ± 3.4
High school or above, n (%)	225 (98.7)
Polypharmacy, n (%)	130 (57.0)
CIRS-G, M (IQR)	5 (3–7)
ADL, M(IQR)	6 (6–6)
IADL, M(IQR)	7 (5–8)
ICOPE screening tool	2 (1–4)
Possible IC decline, n(%)	181 (79.4)
IC detailed assessment	1 (0–2)
Actual IC decline, n(%)	167 (73.2)

*Abbreviations*: *SD* Standard deviation, *BMI* Body mass index, *CIRS-G* The cumulative illness rating scale for geriatrics, *ADL* Activities of daily living, *ICOPE* Integrated care for older people, *IQR* Interquartile range, *IC* Intrinsic capacity

**Fig. 1:**
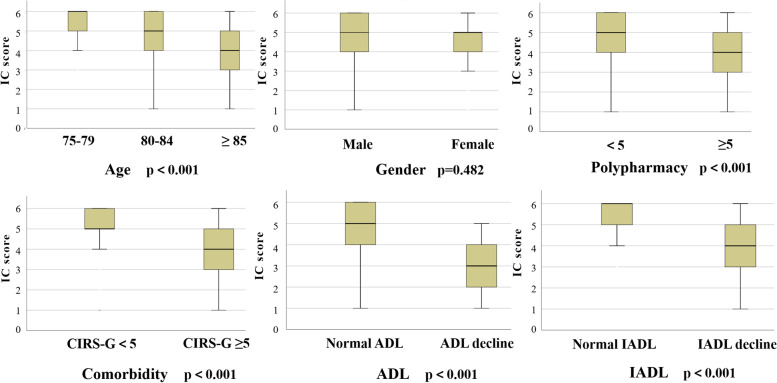
Comparison of the intrinsic capacity scores among different groups in 228 community-dwelling older people. Abbreviations: IC, intrinsic capacity; CIRS-G, the cumulative illness rating scale for geriatrics; ADL, activities of daily living; IADL, instrumental activities of daily living

As shown in Fig. 1, we compared the IC scores of different groups and assessed the differences in IC levels between these groups. The results demonstrated that IC levels decline with increasing age. Older people with polypharmacy, a higher burden of comorbidities, and a decline in ADL exhibited lower levels of IC.

### Intrinsic capacity

In step 1, the ICOPE screening tool was used to identify possible IC decline among older people, and the proportion identified was 79.4%. The proportions of participants with impaired locomotion, vitality, cognition, psychology, vision, and hearing were 50.0%, 13.2%, 53.9%, 35.1%, 19.7%, and 20.2%, respectively. In Step 2, a detailed evaluation method was used to assess the proportion of older people with actual IC decline (73.2%). The proportions of patients with impaired locomotion, vitality, cognition, and psychology were 60.1%, 17.1%, 17.1%, and 16.2%, respectively (Table 3).

**Table 3:** IC decline and impairment of each domain in 2-step assessment

	Step1 ICOPE screening tool	Step 2 detailed IC assessments
IC decline, n (%)	181 (79.4)	167 (73.2)
IC domain impairment		
Locomotion, n (%)	114 (50.0)	137 (60.1)
Vitality, n (%)	30 (13.2)	39 (17.1)
Cognition, n (%)	123 (53.9)	39 (17.1)
Psychology, n (%)	80 (35.1)	37 (16.2)
Sensory (vision), n (%)	45 (19.7)	45 (19.7)
Sensory (hearing), n (%)	46 (20.2)	46 (20.2)

*Abbreviations*: *IC* Intrinsic capacity, *ICOPE* Integrated care for older people

### Validity of the ICOPE screening tool

**Fig. 2:**
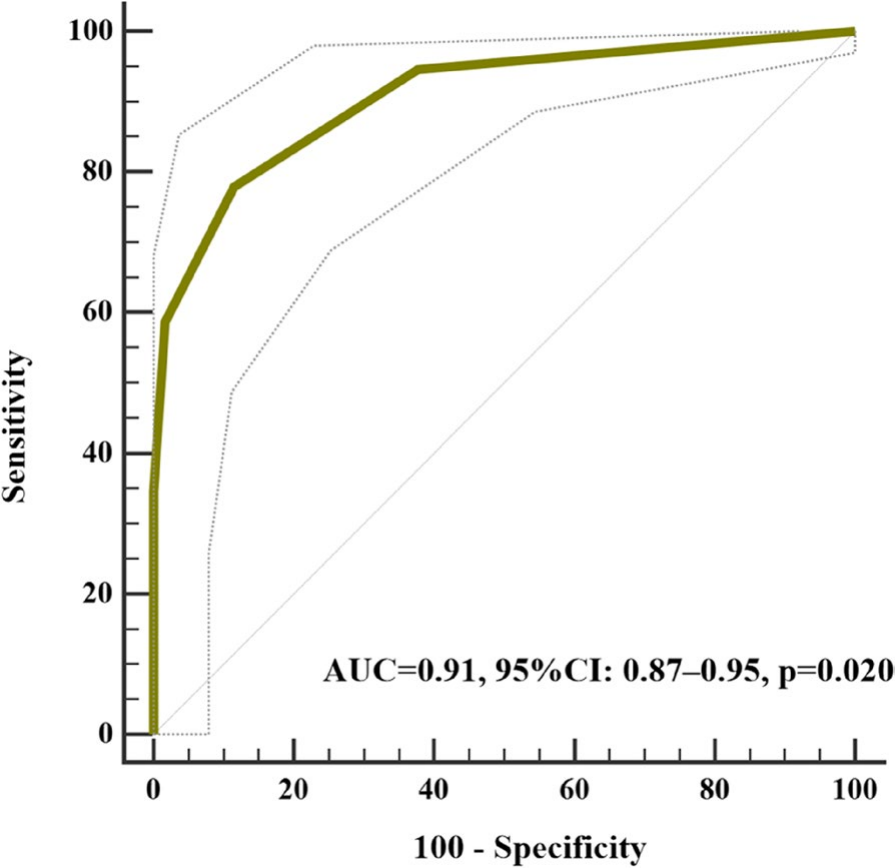
Diagnostic effectiveness of the ICOPE screening tool. Abbreviations: ICOPE, integrated care for older people; AUC, areas under the curve; CI, confidence interval

The ICOPE screening tool had a sensitivity and specificity of 94.6% and 62.3% for identifying IC decline, respectively, with a diagnostic accuracy of 86.0%. Except for the sensory dimension, the sensitivity for detecting impairment in each dimension of the IC using the ICOPE screening tool was the highest for cognition (100%) and the lowest for vitality (51.3%). Regarding specificity, the vitality dimension performed the best at 94.7%, whereas cognition had the lowest specificity at 55.6% (Table 4). As shown in Fig. 2, the diagnostic effectiveness of the ICOPE screening tool was 0.91 (95% confidence interval: 0.87–0.95, *p* = 0.020).

**Table 4:** Sensitivity, specificity and diagnostic accuracy of ICOPE screening tool

Detailed IC assessment (IC domain impairment)		Possible IC decline by ICOPE screening tool		Sensitivity (%)	Specificity (%)	Diagnostic accuracy (%)
	Assessment result	Yes	No			
IC decline	Yes	158	9	94.6	62.3	86.0
	No	23	38			
Locomotion	Yes	108	29	78.8	93.4	84.6
	No	6	85			
Vitality	Yes	20	19	51.3	94.7	87.3
	No	10	179			
Cognition	Yes	39	0	100	55.6	63.2
	No	84	105			
Psychology	Yes	27	10	73.0	72.3	72.4
	No	53	138			

*Abbreviations*: *IC* Intrinsic capacity, *ICOPE* Integrated care for older people

Body mass index (BMI) < 20 kg/m^2^ [22] was added to the original ICOPE screening tool for vitality dimension to improve the sensitivity of vitality dimension screening. The vitality domain may be impaired if any of the conditions in the vitality screening question are met. As shown in Table 5, the sensitivity and specificity of the modified ICOPE screening tool were 95.2% and 55.7%, respectively, and the sensitivity of the vitality domain screening increased to 69.2%, with a specificity and diagnostic accuracy of 90.5% and 86.8%, respectively.

**Table 5:** Sensitivity, specificity and diagnostic accuracy of modified ICOPE screening tool

Detailed IC assessment (IC domain impairment)		Possible IC decline by ICOPE screening tool		Sensitivity (%)	Specificity (%)	Diagnostic accuracy (%)
	Assessment result	Yes	No			
IC decline	Yes	159	8	95.2	55.7	84.6
	No	27	34			
Vitality	Yes	27	12	69.2	90.5	86.8
	No	18	171			

*Abbreviations*: *IC* Intrinsic capacity, *ICOPE* Integrated care for older people

## Discussion

This study verified the feasibility of the ICOPE screening tool among community-dwelling older people in China based on the WHO proposed assessment pathway for IC in older people. The ICOPE screening tool demonstrated a high sensitivity, specificity, and diagnostic accuracy of 94.6%, 62.3%, and 86.0% in identifying older people with IC decline, respectively. It can be used as a simple and effective screening tool for promotion and application in the community.

The ICOPE screening tool showed varying diagnostic effectiveness for different IC dimensions. The highest sensitivity was observed in the cognitive domain, reaching 100%, whereas the lowest sensitivity was noted in the vitality domain, at 51.3%. A possible reason for the poor sensitivity in vitality is that, in the nutrition screening question, weight loss is defined as “unintentional weight loss ≥ 3 kg in the past 3 months”, while some older people may have lost ≤ 3 kg. Furthermore, for some underweight older people, weight loss may not be significant; however, they may still be at risk of malnutrition. These factors may increase the false-negative rate of the screening tool, leading to a decrease in sensitivity.

Given that a screening tool should have high sensitivity, one method to improve the sensitivity is to lower the weight threshold in the vitality dimension screening question, although the specific value requires further research and discussion. Moreover, adding BMI screening to the screening questions can increase sensitivity. Based on the Global Leadership Initiative on Malnutrition diagnostic criteria recommended for Asians [22] aged ≥ 70 years, a BMI < 20 kg/m^2^ indicates BMI reduction. In this study, the proportion of older adults with a BMI reduction was 10.1% (23 cases). By including BMI reduction in nutritional screening, the proportion of older adults who may be at risk of malnutrition increased to 19.7% (45 cases). Using the short-form mini-nutritional assessment as the gold standard, the sensitivity of the vitality screening tool increased to 69.2% with a specificity of 90.5% (Table 5). This study provides a reference for future improvements in the ICOPE screening tools. Additionally, the screening question used for the locomotion domain in the ICOPE screening tool was whether the time for the five-time sit-to-stand test (FTSST) was ≥ 14 s. This study mainly included older people of advanced age. We used a short physical performance battery score of ≤ 9 points as the gold standard for impairment in the locomotion domain. Receiver operating characteristic analysis was used to determine that the optimal threshold for the FTSST in this study population was 13.9 s, which was consistent with the recommended threshold in the ICOPE screening tool. However, this threshold may be low in the young age of older people, leading to a decrease in sensitivity. Different age groups may have different FTSST thresholds. Therefore, further sample expansion is required to explore and establish appropriate screening thresholds for the locomotion domain.

The cognitive domain had the lowest specificity among all screening tools (55.6%). In this study, among older people with abnormal cognitive screening results, 95.9% had a decrease in word recall ability, while only 39.0% had impairments in time and space orientation. This suggests that many older people may only experience a decline in word recall ability, whereas memory decline is common in older people and may be unrelated to cognitive impairment. This may be a reason for the low specificity of the cognitive dimension-screening tool.

A study in Spain [12] enrolled 207 older people aged ≥ 70 years, based on the WHO IC assessment pathway, including screening and detailed evaluation of vision and hearing. The results revealed that among all dimensions, the cognitive domain had the highest sensitivity (0.889), with the other dimensions ranging from 0.438–0.557. Among them, vision (0.438), hearing (0.452), and vitality (0.455) domains showed poor sensitivity. The specificities of all dimensions were reasonable, with the vitality domain being the best (0.960), and that of the other domains ranging from 0.682 to 0.953. Overall, their results were similar to those of our study, and some dimensional issues of the ICOPE screening tool require improvement. Another study [11] conducted in Hong Kong, China, included 304 community residents aged ≥ 60 years and found that the sensitivity and specificity of the ICOPE screening tool were 95.0% and 57.6%, respectively. This is consistent with the results of our study. However, the sensitivity of each IC dimension was the highest in the vitality domain, reaching 100%. This difference may be because the study population was relatively younger, and there may have been fewer weight loss individuals, with a lower proportion of actual malnutrition risk or malnourished individuals and a lower false-negative rate.

In this study, 181 (79.4%) older people exhibited a possible IC decline when screened using the ICOPE screening tool; 167 (73.2%) had actual IC decline when assesses using detail assessment. Ma et al. [23] assessed 376 hospitalized older people (68.7 ± 11.4 years) using the ICOPE screening tool and found that 260 (69.1%) individuals exhibited a possible IC decline. Other studies have reported varying proportions of IC decline ranging from 43.0–64.5% [5, 24]. The higher proportion of IC decline in this study may be attributed to the older age of the participants. This study also found that older age, polypharmacy, a higher burden of comorbidities, and a decline in ADL were associated with lower IC levels. These findings are consistent with the results reported by Ma et al. [23]. Additionally, Christine et al. [25] found that cognitive, visual, and hearing impairments were independently associated with ADL decline based on data from the Health and Retirement Study. Furthermore, Sarwat et al. [26] reported that impairments in locomotion (hazard ratio [HR] = 3.03, *p* < 0.001), cognition (HR = 1.62, *p* < 0.001), vision (HR = 1.52, *p* < 0.001), and mental health (HR = 1.71, *p* < 0.001) were independently associated with an increased risk of ADL decline based on data from the Cardiovascular Health Study with a 7-year follow-up. These studies collectively indicate a close relationship between IC levels and functional status in older people.

### Strength and limitation

IC decline is commonly observed in older people and is associated with adverse outcomes [27–32]. Adopting a screening-before-assessment model can utilize community resources more efficiently. The main strength of this study is that few studies have validated the diagnostic performance of the ICOPE screening tool. This enriches the research foundation in this field, provides theoretical references for promoting the ICOPE screening tool in the community, and suggests methods to improve its sensitivity. However, this study also has some limitations. First, this was a single-center, small-sample study, and further sample expansion is needed to validate and extrapolate the conclusions. Second, the screening and detailed evaluation of sensory dimensions in this study were both self-assessment questionnaires. However, considering the limitations of community resources, the evaluation of sensitivity and specificity for sensory screening may be insufficient. Therefore, objective indicators should be used for further evaluation of the sensory domain in future studies.

## Conclusion

The ICOPE screening tool has high sensitivity and can be used as an IC screening scale in community-dwelling older people. However, further improvements are needed in the vitality domain of the ICOPE screening tool to enhance its sensitivity in identifying individuals at risk of malnutrition.

## Data Availability

The datasets analyzed in the current study are available from the corresponding author upon request.

## Abbreviations

ADL: Activities of daily living
BMI: Body mass index
CIRS-G: The cumulative illness rating scale for geriatrics
FTSST: Five-time sit-to-stand test
GDS: Geriatric depression scale
ICOPE: Integrated care of older people
IC: Intrinsic capacity
IADL: Instrumental activities of daily living
MMSE: Mini-mental state examination
MNA-SF: Short-form mini-nutritional assessment
PSMS: Physical self-maintenance scale
SPPB: Short physical performance battery
WHO: World health organization
